# Voluntary settlement and its consequences on predictors of happiness: the influence of initial cultural context

**DOI:** 10.3389/fpsyg.2014.01311

**Published:** 2014-11-17

**Authors:** Keiko Ishii, Shinobu Kitayama, Yukiko Uchida

**Affiliations:** ^1^Department of Psychology, Kobe UniversityKobe, Japan; ^2^Department of Psychology, University of MichiganAnn Arbor, MI, USA; ^3^Kokoro Research Center, Kyoto UniversityKyoto, Japan

**Keywords:** independence and interdependence, voluntary settlement, happiness, influence/personal behaviors, adjustment/interpersonal behaviors

## Abstract

Hokkaido—a northern island of Japan that was settled by ethnic Japanese during the late nineteenth century and the early twentieth century—may remain to be a hybrid of interdependent culture of the mainland Japan and independent culture associated with frontier settlement. We thus anticipated that contemporary Hokkaido residents would exhibit either independent or interdependent psychological profiles depending on the types of behaviors that were required in a given situation. As expected, happiness was associated with positive disengaging emotions (e.g., pride in the self)—an independent profile—in situations that required personal goal pursuit and interpersonal influence; however, happiness was associated with positive engaging emotions (e.g., feelings of closeness)—an interdependent profile—in situations that required interpersonal harmony and adjustment. In contrast, such situational dependency was not observed for either mainland Japanese or Americans. For mainland Japanese happiness was associated with positive engaging emotions whereas for Americans happiness was associated with positive disengaging emotions.

## Introduction

Over the last two decades substantial effort has been made to investigate cultural variations across broad regions that are glossed as East (encompassing much of Asia) and West (North America and Western Europe) (e.g., Markus and Kitayama, 1991; Nisbett et al., 2001). The growing body of evidence suggests that this broad distinction between East and West can serve as a reasonable first approximation in investigating cultural variations on the globe. It also suggests that independence vs. interdependence (or individualism vs. collectivism) (Triandis, 1989; Markus and Kitayama, 1991) is a key dimension to foster the cultural variations. Nevertheless, few investigations pay attention to a vast amount of variation that is likely to exist within each broad cultural area. Thus, concerted effort toward understanding within-culture variation is very much called for.

It is reassuring, then, that over the last several years researchers have examined a number of variables that may be linked to within-culture variations including social class (e.g., Snibbe and Markus, 2005; Kraus et al., 2009), power (e.g., Miyamoto and Wilken, 2010), residential mobility (e.g., Oishi, 2010), daily economic activities (e.g., Uskul et al., 2008), urbanism (e.g., Kashima et al., 2004; Yamagishi et al., 2012), and infectious disease prevalence (Fincher et al., 2008). One such variable that may prove to be particularly important in understanding the nature of new cultural forms when people migrate new lands of frontier and initiate their society is frontier settlement. In the present work, we will focus on Hokkaido—a northern island of Japan that was settled by ethnic Japanese during the second half of the nineteenth century and the first half of the twentieth and examine the hypothesis that contemporary culture of Hokkaido is a hybrid of culture of mainland Japan (which features interdependence) and culture of frontier (which is likely to be dominated by needs of, as well as desires for, independence).

### Hokkaido: japan's northern frontier

Hokkaido is located at the northern Japan, close to Russia, where an indigenous people called Ainu formed a hunter-gathering society. It was a wilderness area until the middle of the nineteenth century. After that, the new Meiji government sent numerous jobless Samurai warriors into Hokkaido to settle and take control over Hokkaido and prepare defense against a perceived threat from Russia. This governmental initiative was followed by the migration of numerous farmers and peasants, who voluntarily moved to Hokkaido to settle and to seek land. Immigration continued throughout the next several decades, transforming Hokkaido into an integral part of Japan with a population of nearly three million by the middle of the twentieth century. Thus, Hokkaido is similar to North American in terms of the history of voluntary settlement.

Voluntary settlement is a factor generating the ethos of independence, which consists of collective beliefs and practices of independent agency (Turner, 1920). According to the voluntary settlement hypothesis (Kitayama et al., 2009; Kitayama and Bowman, 2010), frontier ecological conditions are usually harsh and social institutions are often primitive; consequently, frontier settlers are more likely to develop a sense of independent agency, which is represented in orientation toward personal goal pursuits and personal choice. Supporting the hypothesis, Kitayama et al. (2009) demonstrated that North Americans were quite independent compared to Western European groups of the UK and Germany, assessed by several measurements including dispositional attribution and symbolic self-inflation. In contrast, mainland Japanese were less independent than North Americans and the two groups of Western European.

Similar effect of voluntary settlement would be expected in Hokkaido sharing the history of voluntary settlement with North America. Indeed, Kitayama et al. (2006a) demonstrated that Hokkaido-born Japanese in Hokkaido are more similar to North Americans than to mainland Japanese in terms of three indicators of independent agency (personal goal pursuits, personal choice, and the dispositional lay theory of social behavior). Moreover, Yamawaki (2012), which created an index of Japanese collectivism, showed that Hokkaido is the second lowest of the 47 prefectures of Japan in terms of the index. Only Tokyo, the largest metropolitan city of Japan, exceeded Hokkaido. This suggests that not only urbanism (e.g., Kashima et al., 2004; Yamagishi et al., 2012) but also the history of voluntary settlement would contribute to the regional variation in the level of collectivism.

However, North America and Hokkaido are very different in terms of the initial cultural context in which voluntary settlement occurred. While settlers of North America were mostly Western European origin, settlers of Hokkaido were ethnic Japanese. Voluntary settlement in Hokkaido is thus unique because it occurred in the context of the predominant mainland-Japanese ethos, which is embedded in a larger cultural context of interdependence. This raises an important research question whether in Hokkaido the idea of interdependence fostered by the predominant mainland-Japanese ethos is likely to coexist with the idea of independence generated by voluntary settlement.

Culture is conceptualized as a collective-level phenomenon that is composed of both socially shared meanings such as ideas and beliefs and scripted behavioral patterns of norms and practices (Kitayama and Uskul, 2011). It can evoke and guide different sets of psychological responses. If independence and interdependence coexist in Hokkaido, they would facilitate the corresponding psychological responses, that is, a tendency toward personal goal pursuit fostered by independence and a tendency toward achievement of interpersonal harmony fostered by interdependence. In addition, these psychological tendencies would emerge differently depending on social situations in which people are involved. As evidence related to such a situational influence, cultural priming studies for biculturally socialized individuals have shown that cultural knowledge of ideas and beliefs, which are activated by such “primed” linguistic symbols and icons, guides the corresponding psychological tendencies (e.g., Hong et al., 2000).

Unfortunately, the possibility on switching between the two tendencies in Hokkaido people was not tested in Kitayama et al. (2006a). For example, in their first study, because the participants were asked to describe the most emotional episode they had recently experienced, the types of emotional situations experienced were not manipulated. Thus, the result that as with happiness for the North American participants, happiness for the Hokkaido Japanese participants was distinctly more personal might appear because these Hokkaido Japanese participants were likely to incidentally remember emotional situations fostering the orientation toward the idea of independence. In the present research we examined the untested hypothesis that contemporary residents of Hokkaido would be “bi-cultural,” in the sense that they show both tendencies toward personal goal pursuit and achievement of interpersonal harmony depending on the specific goals that are emphasized in given social situations, focusing on predictors of happiness.

### Cultural differences in predictors of happiness

Past studies have shown that what predicts happiness differs across cultures, corresponding to the ideas of independence and interdependence (e.g., Oishi and Diener, 2001). Happiness is associated with personal achievement in Western cultures where the idea of independence is spread, whereas happiness is associated with social harmony in East Asian cultures where the idea of interdependence is dominant. For instance, Kitayama et al. (2000, 2006b) demonstrated that the general positive emotional experiences (e.g., happiness) reported by North Americans are strongly associated with emotional experiences related to the accomplishment of independence (e.g., self-esteem and pride in self), which are known as disengaging positive emotions. In contrast, they demonstrated that the general positive emotional experiences reported by Japanese are strongly associated with emotional experiences related to an accomplishment of interdependence (e.g., respect and friendly feelings), which are termed engaging positive emotions.

The general patterns of North Americans and Japanese were replicated by Kitayama et al. (2006a). They asked four groups of participants (North Americans, Hokkaido-born Japanese in Hokkaido, mainland-born Japanese in Hokkaido, and mainland Japanese) to briefly describe the most emotional episode they had recently experienced and to indicate how strongly they had experienced a list of emotions presented. The reported strength of generally positive emotions was more strongly predicted by that of disengaging positive emotions than that of engaging positive emotions in North Americans, whereas it was more strongly predicted by that of engaging positive emotions than that of disengaging positive emotions in mainland Japanese. Moreover, the patterns of the two Hokkaido groups fell between those of North American and mainland Japanese groups. Although the reported strength of generally positive emotions was predicted by both disengaging and engaging positive emotions in Hokkaido-born Japanese in Hokkaido, their pattern was similar to that of North Americans, but different from that of mainland Japanese. While the reported strength of generally positive emotions was more strongly predicted by engaging positive emotions than disengaging positive emotions in mainland-born Japanese in Hokkaido, the association between generally positive emotions and disengaging positive emotions was significantly stronger compared to that in mainland Japanese. In addition, no significant difference between the patterns of the two Hokkaido groups was found. These results suggest that the idea of independence, encouraged by the history of voluntary settlement, underlies predictors of one's happiness in Hokkaido. However, given the difference between North America and Hokkaido in cultural heritage where the settlement occurred, the cultural differences in predictors of happiness might be moderated by the types of behaviors that were required in a given situation.

### The present research

We conducted two studies to test the predictions that the ideas of independence and interdependence coexist in contemporary resident of Hokkaido, and that whether happiness is better predicted by either disengaging positive emotions or engaging positive emotions depends on social situations they experience. In Study 1, we asked participants to report the intensity of experiencing emotions in a preselected set of situations, including personal and interpersonal behaviors. In Study 2, we asked participants to report how intensely they experienced a number of emotions in each of the influence and adjustment situations they remembered. In both studies, we examined the associations between general positive emotions and disengaging/engaging positive emotions for each of the types of situations.

Personal goal pursuit and interpersonal influence are emphasized when independent agency is highly valued (Morling et al., 2002). People are motivated toward behaviors related to personal goal and influence in social environments and situations where the idea of independence prevails. They will feel disengaging positive emotions such as self-esteem and pride by accomplishing a personal achievement and then experience happiness. On the other hand, interpersonal harmony and adjustment are emphasized when interdependence is highly valued (Morling et al., 2002). People are motivated toward behaviors related to adjustment and harmony in social environments and situations where the idea of interdependence is widespread. They will feel engaging positive emotions such as respect and friendly feelings by accomplishing an interpersonal goal including adjustment and harmony and then experience happiness. If happiness of contemporary Hokkaido residents depends on which of the ideas is emphasized in a given social situation, it may be likely to link to disengaging positive emotions in social situations where the idea of independence is highly valued (e.g., personal and influence situations). In contrast, their happiness may be likely to link to engaging positive emotions in social situations where the idea of interdependence is highly valued (e.g., interpersonal and adjustment situations).

As in Kitayama et al. (2006a), we tested groups of North Americans, Hokkaido-born Japanese in Hokkaido, mainland-born Japanese in Hokkaido, and mainland Japanese. This enabled us to show cultural differences regarding the association between happiness and disengaging/engaging positive emotions that may be moderated by the types of situations. Given the previous findings by Morling et al. (2002), if behaviors related to influence and efficacy are highly valued in North Americans, the association between happiness and disengaging positive emotions may become particularly strong when a personal goal is achieved (e.g., influence situations). In contrast, if behaviors related to adjustment and harmony are highly valued in mainland Japanese, the association between happiness and engaging positive emotions may become particularly strong when an interpersonal goal is achieved (e.g., adjustment situations).

Moreover, Kitayama et al. (2006a) found that mainland-born Japanese in Hokkaido was not statistically different from Hokkaido-born Japanese in Hokkaido in terms of the degree of an association between happiness and disengaging positive emotions and that of an association between happiness and engaging ones. They interpreted the result based on the possibility of selection bias. That is, a minority of mainland-born Japanese who are highly motivated toward personal goal pursuit might have psychological sympathy to and be attracted to Hokkaido. The possibility suggests that this group might represent contemporary settlers who moved voluntarily to Hokkaido. In contrast, differences on their heritage culture whether they were born and brought up either in Hokkaido or in mainland Japan might influence how solid their independent tendencies are. Thus, the similarity between the two Hokkaido groups might be replicated in the current study. At the same time, however, situational effects on personal goal pursuit might be weaker in mainland-born Hokkaido Japanese compared to Hokkaido-born Hokkaido Japanese.

In sum, we hypothesized that (1) in Hokkaido-born Japanese in Hokkaido, happiness would be associated with disengaging positive emotions in social situations where the idea of independence is highly valued, whereas happiness would be associated with engaging positive emotions in social situations where the idea of interdependence is highly valued, (2) in North Americans, happiness would be associated with disengaging positive emotions, particularly in social situations where the idea of independence is highly valued, (3) in mainland Japanese, happiness would be associated with engaging positive emotions, particularly in social situations where the idea of interdependence is highly valued, and (4) while the pattern on predictors of happiness in mainland-born Japanese in Hokkaido might be similar to that in Hokkaido-born Japanese in Hokkaido, the situational influences might be weak in mainland-born Japanese in Hokkaido. We tested these hypotheses together. Following Simmons et al. (2012)'s recommendation, we report how we determined our sample size, all data exclusions (if any), all manipulations, and all measures in the present research.

## Study 1

### Method

Eighty Hokkaido-born Japanese students (31 females and 49 males, *M*_age_ = 18.63, *SD* = 1.35) and 68 mainland-born Japanese students (15 females and 53 males, *M*_age_ = 18.53, *SD* = 0.72) at Hokkaido University^1^, 475 mainland-born Japanese students (164 females and 311 males) at Kyoto University^2^, and 59 students (33 females and 26 males, *M*_age_ = 19.97, *SD* = 1.36) at the University of Michigan participated in the study for partial course credit or monetary compensation. The mainland-born Hokkaido Japanese participants' mean length of stay in Hokkaido was 5.31 months (*SD* = 11.93). This study was approved by the Ethics Committee of the Center for Experimental Research in Social Sciences at Hokkaido University. All participants filled out the Implicit Social Orientation Questionnaire (ISOQ) (Kitayama and Park, 2007; Kitayama et al., 2009). In the ISOQ, participants are presented with 6 situations related to personal behaviors and 4 situations related to interpersonal behaviors (see Table 1). Participants were asked to remember the last event pertaining to each of the 10 situations. Then, they were to report the extent to which they experienced a series of emotions in each of the situations on a 6-point rating scale (1 = not at all, 6 = very strongly). The emotions used were adopted from previous studies by Kitayama and Park (2007; Kitayama et al., 2009). Emotions were sampled from six theoretically derived categories. Some emotions were general in the sense that they could be associated with either independence or interdependence, both positive (happy, elated, and calm) and negative (unhappy). Others were socially disengaging, resulting from either success (proud and self-esteem) or failure with regard to independence (angry and frustration). Still others were socially engaging, stemming from either success (friendly feelings and close feelings) or failure with regard to interdependence (ashamed and guilty).

**Table 1:** **Ten situations involved in the ISOQ (Study 1)**.

**Personal**
Situation 1: When you thought about your appearance
Situation 2: When you read a novel or book
Situation 3: When you watched TV or listened to music
Situation 4: When you got ill or injured
Situation 5: When you were caught in a traffic jam
Situation 6: When you were overloaded with work
**Interpersonal**
Situation 1: When you had positive interaction with friends
Situation 2: When you had good interaction with a family member
Situation 3: When something good happened to a family member of yours
Situation 4: When you had a problem with a family member

### Results and discussion

The average intensity for each participant was computed for general positive emotions, disengaging positive emotions, and engaging positive emotions for each situation. To see situational influences in each culture, the mean standardized regression coefficients predicting happiness for disengaging and engaging positive emotions for each situation in each culture were computed. They are shown in Table 2. We then compared the degrees of the two standardized regression coefficients for each situation in each culture following a procedure explained in Steiger (1980). As shown in Table 2, regression coefficients for engaging positive emotion were significantly larger in mainland Japanese than those for disengaging positive emotion regardless of the situations, except for one situation (situation 1, personal behavior). In contrast, no significant difference was observed between the degrees of the two regression coefficients for North Americans, except for one situation. In the exceptional situation (situation 4, interpersonal behavior), the regression coefficient for disengaging positive emotion was significantly larger than that for engaging positive emotion. Notably, regression coefficients for engaging positive emotion in the two Hokkaido groups were significantly larger than those for disengaging positive emotion in some of the interpersonal situations. This tendency was similar to that in mainland Japanese. Unlike for mainland Japanese, however, for the two Hokkaido groups there was no difference in the degrees of the two regression coefficients for disengaging positive emotions and engaging positive emotions in the personal situations, except for one situation for mainland-born Japanese in Hokkaido (situation 5). Rather, this tendency was similar to that in North Americans^3^.

**Table 2:** **Standardized regression coefficients predicting happiness for disengaging and engaging positive emotions, which were computed for each situation in each culture (Study 1)**.

	**DEP**	**EP**	***t***	***p***	***r*(effect-size)**
**NORTH AMERICANS**					
**Personal**					
Situation 1	0.41^**^	0.36^**^	0.22	0.83	0.03
Situation 2	0.35^**^	0.41^**^	−0.28	0.78	0.04
Situation 3	0.29^*^	0.36^**^	−0.28	0.78	0.04
Situation 4	0.42^**^	0.19	1.35	0.18	0.18
Situation 5	0.06	0.24	−0.76	0.45	0.10
Situation 6	0.39^**^	0.34^**^	0.27	0.79	0.04
**Interpersonal**					
Situation 1	0.37^**^	0.43^**^	−0.37	0.71	0.05
Situation 2	0.37^**^	0.39^**^	−0.0.9	0.93	0.01
Situation 3	0.46^**^	0.36^**^	0.53	0.60	0.07
Situation 4	0.43^**^	0.04	2.07	<0.05	0.27
**HOKKAIDO-BORN JAPANESE IN HOKKAIDO**					
**Personal**					
Situation 1	0.52^**^	0.39^**^	0.86	0.39	0.10
Situation 2	0.19	0.27^*^	−0.43	0.67	0.05
Situation 3	0.28^**^	0.37^**^	−0.47	0.64	0.05
Situation 4	0.45^**^	0.47^**^	−0.11	0.91	0.01
Situation 5	0.26	0.32^*^	−0.20	0.84	0.03
Situation 6	0.44^**^	0.31^**^	0.77	0.44	0.09
**Interpersonal**					
Situation 1	0.29^**^	0.68^**^	−3.53	<0.001	0.37
Situation 2	0.13	0.58^**^	−2.88	<0.01	0.31
Situation 3	0.45^**^	0.53^**^	−0.61	0.54	0.08
Situation 4	0.05	0.69^**^	−4.19	<0.0001	0.49
**MAINLAND-BORN JAPANESE IN HOKKAIDO**					
**Personal**					
Situation 1	0.53^**^	0.34^**^	1.07	0.29	0.13
Situation 2	0.24^*^	0.33^**^	−0.54	0.59	0.07
Situation 3	−0.03	0.31^*^	−1.52	0.13	0.19
Situation 4	0.22^*^	0.42^**^	−1.22	0.23	0.15
Situation 5	−0.07	0.82^**^	−5.17	<0.0001	0.64
Situation 6	0.43^**^	0.54^**^	−0.82	0.42	0.10
**Interpersonal**					
Situation 1	0.08	0.78^**^	−5.23	<0.0001	0.54
Situation 2	−0.09	0.66^**^	−5.20	<0.0001	0.54
Situation 3	0.47^**^	0.34^**^	0.71	0.48	0.10
Situation 4	0.25	0.22	0.16	0.87	0.02
**MAINLAND JAPANESE**					
**Personal**					
Situation 1	0.46^**^	0.37^**^	1.48	0.14	0.07
Situation 2	0.23^**^	0.41^**^	−2.61	<0.01	0.12
Situation 3	0.18^**^	0.45^**^	−3.83	<0.001	0.17
Situation 4	0.28^**^	0.43^**^	−2.25	<0.05	0.10
Situation 5	0.19^**^	0.47^**^	−3.95	<0.0001	0.18
Situation 6	0.34^**^	0.51^**^	−2.70	<0.01	0.12
**Interpersonal**					
Situation 1	0.16^**^	0.67^**^	−9.70	<0.0001	0.41
Situation 2	0.08^**^	0.78^**^	−14.71	<0.0001	0.56
Situation 3	0.29^**^	0.59^**^	−5.53	<0.0001	0.25
Situation 4	0.21^**^	0.51^**^	−4.81	<0.0001	0.22

*DEP, disengaging positive emotion; EP, engaging positive emotion*.

** *p < 0.01*,

* *p < 0.05*.

For each situation we also performed a multiple regression analysis including the culture and disengaging positive emotion and the culture and engaging positive emotion interactions to see cultural differences in the associations between happiness and disengaging positive emotions and between happiness and engaging positive emotions. The results are summarized in Table 3. Given that regression coefficients for engaging positive emotion were larger in mainland Japanese than those for disengaging positive emotion regardless of the situations, whereas no significant difference was observed between the degrees of the two regression coefficients for North Americans, it would be expected that the association between happiness and engaging positive emotions was greater in mainland Japanese than in North Americans. The results fit with the expectation in more than half of the situations (3 out of the 6 personal situations and 3 out of the 4 interpersonal situations including marginally significant interaction effects). Moreover, the association between happiness and disengaging positive emotions tended to be greater in North Americans than in mainland Japanese particularly in the interpersonal situations.

**Table 3:** **Cultural differences in the associations between happiness and disengaging positive emotions and between happiness and engaging positive emotions for each situation (Study 1)**.

	**Culture × DEP**		**Culture × EP**	
	***t***	***p***	***t***	***p***
**CULTURE: NORTH AMERICANS = 1, MAINLAND JAPANESE = 0**				
**Personal**				
Situation 1	−1.00	0.31	−0.62	0.53
Situation 2	0.26	0.79	0.15	0.87
Situation 3	0.34	0.73	−0.96	0.33
Situation 4	0.77	0.44	−1.71	0.09
Situation 5	−1.51	0.13	−1.91	0.06
Situation 6	0.32	0.74	−1.77	0.08
**Interpersonal**				
Situation 1	1.38	0.16	−2.95	<0.01
Situation 2	2.36	<0.05	−4.41	<0.0001
Situation 3	1.76	0.08	−1.27	0.20
Situation 4	1.76	0.08	−5.17	<0.0001
**CULTURE: NORTH AMERICANS = 1, HOKKAIDO-BORN JAPANESE IN HOKKAIDO = 0**				
**Personal**				
Situation 1	−2.11	<0.05	−0.94	0.34
Situation 2	0.30	0.76	0.57	0.57
Situation 3	−0.08	0.93	−0.17	0.86
Situation 4	−1.75	0.08	−2.48	<0.05
Situation 5	−1.07	0.28	−0.53	0.59
Situation 6	−0.46	0.64	−0.10	0.92
**Interpersonal**				
Situation 1	−0.37	0.71	−2.37	<0.05
Situation 2	0.55	0.58	−2.18	<0.05
Situation 3	−0.11	0.91	−1.04	0.30
Situation 4	2.38	<0.05	−5.39	<0.0001
**CULTURE: NORTH AMERICANS = 1, MAINLAND-BORN JAPANESE IN HOKKAIDO = 0**				
**Personal**				
Situation 1	−1.36	0.17	−0.33	0.74
Situation 2	−0.11	0.91	0.56	0.57
Situation 3	1.23	0.21	−0.07	0.94
Situation 4	0.94	0.34	−0.73	0.46
Situation 5	0.68	0.50	−3.40	<0.005
Situation 6	−0.11	0.90	−1.44	0.15
**Interpersonal**				
Situation 1	1.24	0.21	−2.77	<0.01
Situation 2	2.36	<0.05	−2.24	<0.05
Situation 3	0.65	0.51	0.46	0.64
Situation 4	2.14	<0.05	−0.69	0.49
**CULTURE: HOKKAIDO-BORN JAPANESE IN HOKKAIDO = 1, MAINLAND JAPANESE = 0**				
**Personal**				
Situation 1	1.67	0.09	0.58	0.56
Situation 2	−0.18	0.85	−0.65	0.51
Situation 3	0.52	0.60	−0.95	0.34
Situation 4	2.39	<0.05	1.35	0.17
Situation 5	0.33	0.73	−0.58	0.56
Situation 6	0.95	0.34	−1.47	0.14
**Interpersonal**				
Situation 1	1.83	0.07	0.10	0.92
Situation 2	0.95	0.34	−0.75	0.45
Situation 3	2.36	<0.05	0.03	0.97
Situation 4	−1.22	0.22	2.05	<0.05
**CULTURE: MAINLAND-BORN JAPANESE IN HOKKAIDO = 1, MAINLAND JAPANESE = 0**				
**Personal**				
Situation 1	0.88	0.38	−0.15	0.88
Situation 2	0.31	0.85	−0.64	0.52
Situation 3	−1.33	0.18	−0.90	0.36
Situation 4	−0.42	0.67	−1.06	0.29
Situation 5	−1.60	0.11	2.28	<0.05
Situation 6	0.53	0.59	0.02	0.98
**Interpersonal**				
Situation 1	−0.50	0.61	0.70	0.48
Situation 2	−1.93	0.05	−0.99	0.32
Situation 3	1.10	0.27	−2.30	<0.05
Situation 4	−0.63	0.52	−2.38	<0.05
**CULTURE: HOKKAIDO-BORN JAPANESE IN HOKKAIDO = 1, MAINLAND-BORN JAPANESE IN HOKKAIDO = 0**				
**Personal**				
Situation 1	0.79	0.43	0.58	0.56
Situation 2	−0.34	0.73	−0.05	0.95
Situation 3	1.33	0.18	0.08	0.93
Situation 4	2.56	<0.05	2.26	<0.05
Situation 5	1.78	0.08	−2.63	<0.01
Situation 6	0.43	0.66	−1.30	0.19
**Interpersonal**				
Situation 1	1.54	0.12	−0.44	0.65
Situation 2	1.65	0.10	0.06	0.94
Situation 3	0.88	0.38	1.70	0.09
Situation 4	−0.50	0.61	4.14	<0.0001

*DEP, disengaging positive emotion; EP, engaging positive emotion*.

The patterns of the culture and disengaging positive emotion and the culture and engaging positive emotion interactions were equivocal when the two Hokkaido groups were compared with either North Americans or mainland Japanese. This may suggest that the patterns on the predictors of happiness in the two Hokkaido groups fell between those of North Americans and mainland Japanese. As indirect evidence, when Hokkaido Japanese were compared with North Americans, the culture and engaging positive emotion interaction became relatively negligible in the personal situations, whereas it was still significant in some of the interpersonal situations. In addition, the culture and disengaging positive emotion and the culture and engaging positive emotion interaction patterns were also unclear when the two Hokkaido groups were compared.

Although cultural differences in the patterns on the predictors of happiness were equivocal, the results indicate that in Hokkaido-born Japanese in Hokkaido, a tendency that people feel happier as they experience more disengaging positive emotions is relatively strong in a situation where a personal goal is likely to be activated, whereas a tendency that people feel happier as they experience more engaging positive emotions becomes clear in a situation where an interpersonal goal is likely to be activated. Moreover, the pattern in mainland-born Japanese in Hokkaido was similar to that in Hokkaido-born Japanese in Hokkaido. The similarity between the two Hokkaido groups was thus replicated. Furthermore, happiness was more predicted by engaging positive emotions than disengaging positive emotions for mainland Japanese, whereas no difference between the two regression coefficients was found in North Americans. Interestingly, these patterns appeared regardless of the types of behaviors. This may suggest that the association between happiness and disengaging positive emotions in North Americans and the association between happiness and engaging positive emotions in mainland Japanese are relatively stable across situations and behaviors^4^.

## Study 2

Study 1 provided initial evidence on situational influences in the predictors of happiness in Hokkaido. The purpose of Study 2 was to replicate this by testing the same four groups and using a different set of social behavior aspects. Although overall the results of Study 1 fitted with our expectations, some of them were unexpected. For example, the effect of disengaging positive emotions did not exceed that of engaging positive emotions in the personal situations either for North Americans or for Hokkaido-born Japanese in Hokkaido. In addition, the expected association with happiness in North Americans and mainland Japanese was found not only in culturally matched situations (i.e., personal behaviors for North Americans and interpersonal behaviors for mainland Japanese), but also in culturally unmatched situations (i.e., interpersonal behaviors for North Americans and personal behaviors for mainland Japanese). To test our hypotheses again and further examine these findings, we focused on more culturally specific values in the second study: influence and adjustment.

In Study 2, participants were asked to think of situations in which they influenced or adjusted themselves to the surrounding people and to describe five situations that they had recently experienced. Given the results of Study 1, we expected that in Hokkaido-born Hokkaido Japanese, the association between happiness and disengaging positive emotions would emerge significantly in influence situations as in North Americans, whereas the association between happiness and engaging positive emotions would emerge significantly in adjustment situations as in mainland Japanese. Moreover, these tendencies in North Americans and mainland Japanese might be enhanced in culturally matched situations, compared to culturally unmatched situations. Furthermore, the pattern of mainland-born Hokkaido Japanese might not differ from that of Hokkaido-born Hokkaido Japanese.

### Method

Thirty-seven Hokkaido-born Japanese students (14 females and 23 males, *M*_age_ = 19.46, *SD* = 1.13) and 38 mainland-born Japanese students (10 females and 28 males, *M*_age_ = 19.47, *SD* = 0.99) at Hokkaido University, 54 mainland-born Japanese students at Kyoto University (25 females and 29 males, *M*_age_ = 20.03, *SD* = 2.21), and 47 North American students (36 females and 11 males, *M*_age_ = 20.46, *SD* = 1.52) at the University of Michigan participated in the study for partial course credit or monetary compensation^5^. The mainland-born Hokkaido Japanese participants' mean length of stay in Hokkaido was 11.72 months (*SD* = 9.85). This study was approved by the Ethics Committee of the Center for Experimental Research in Social Sciences at Hokkaido University. About half of the participants in each of the cultures were asked to think of situations in which they had influenced the surrounding people and to describe five situations that they had recently experienced (influence condition). The remaining participants were asked to think of situations in which they had adjusted themselves to the surrounding people and to describe five situations that they had recently experienced (adjustment condition). All the participants were then asked to indicate how strongly they experienced a series of emotions in each situation (1 = not at all, 6 = very strongly). The emotions used were identical to those in Study 1^6^.

### Results and discussion

**Figure 1:**
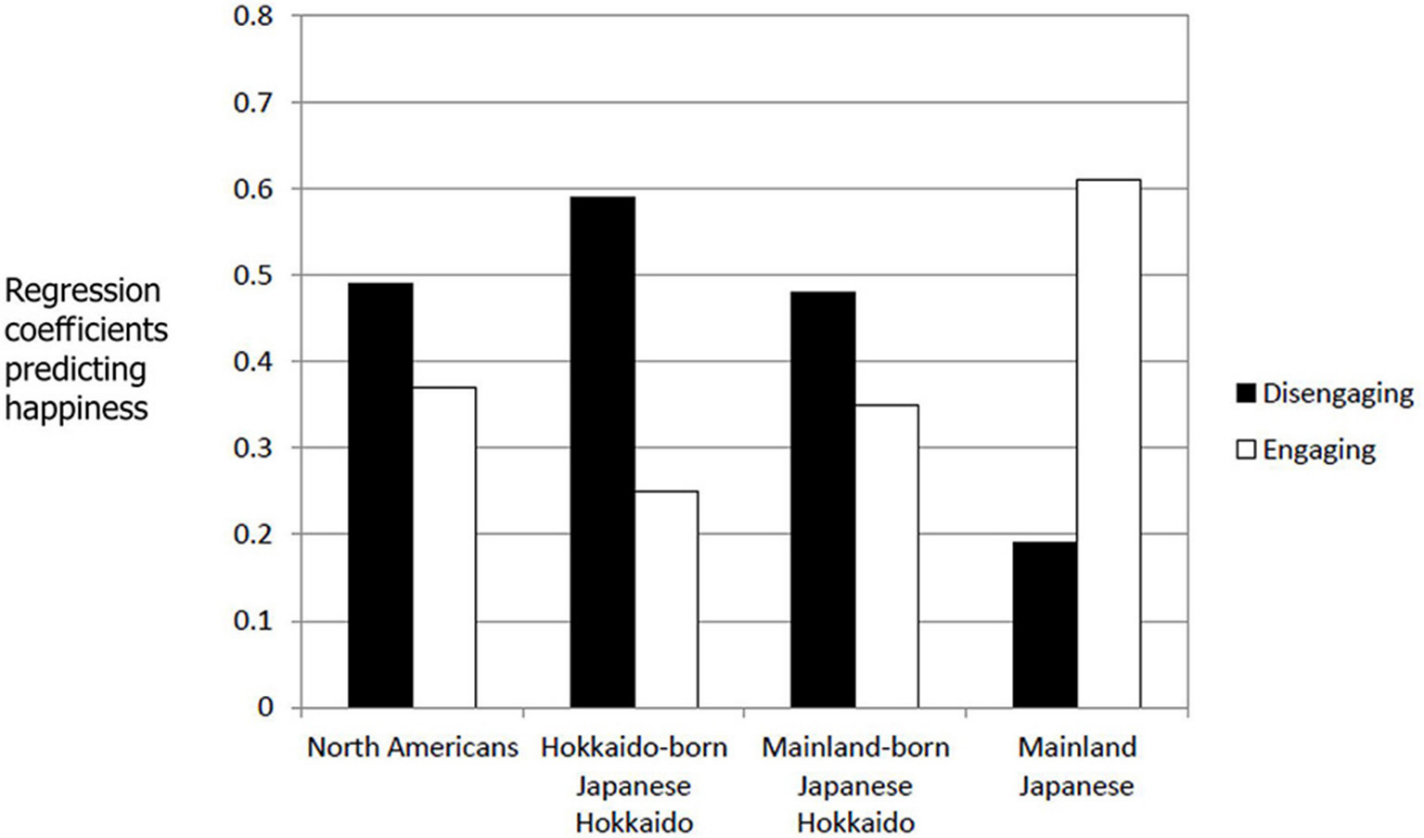
**Standardized regression coefficients predicting happiness for disengaging and engaging positive emotions in the influence condition (Study 2)**.

**Figure 2:**
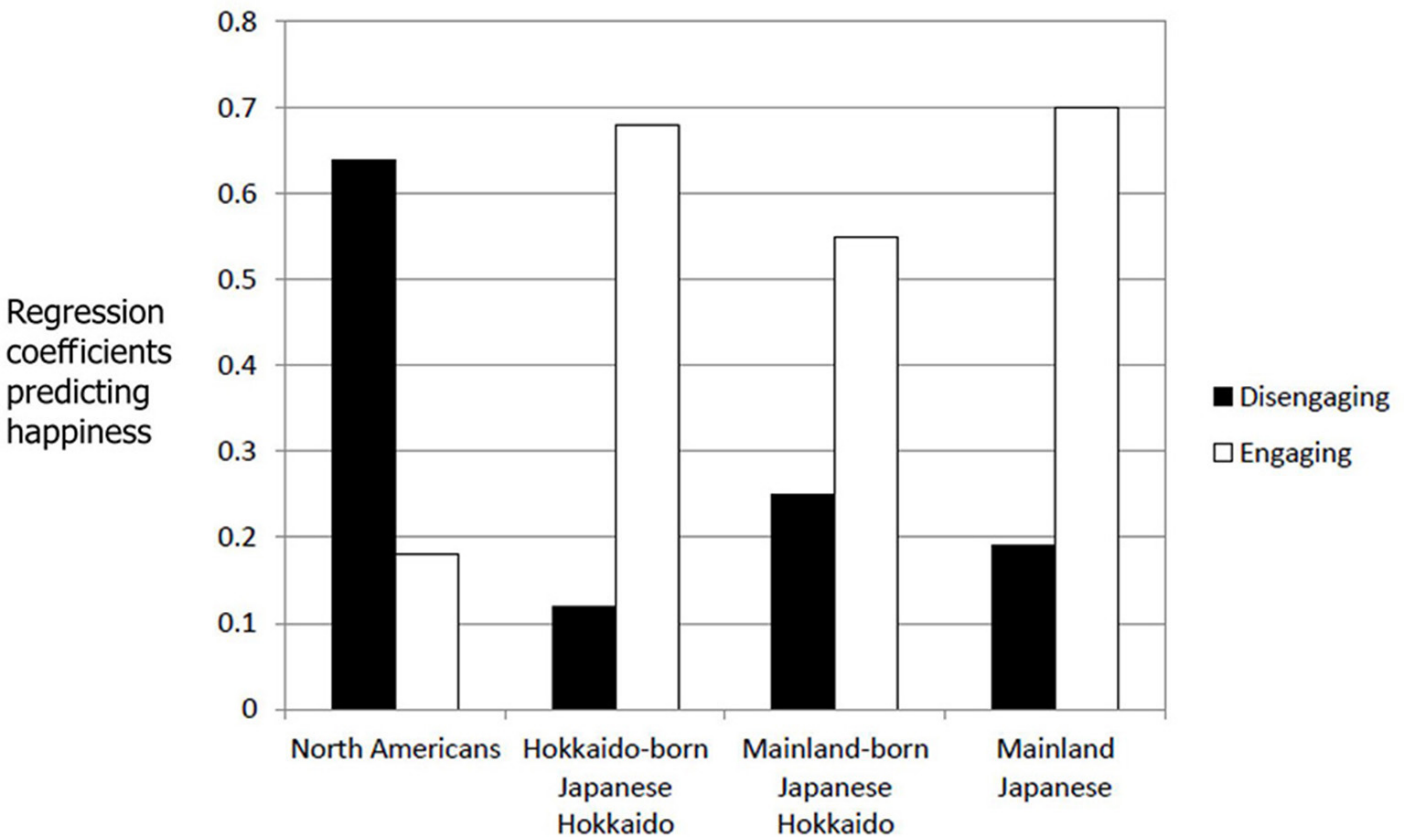
**Standardized regression coefficients predicting happiness for disengaging and engaging emotions positive in the adjustment condition (Study 2)**.

Because unlike in Study 1, participants asked to describe five different situations in each condition, the average intensity ratings of the five situations were computed for each participant for general positive emotions, disengaging positive emotions, and engaging positive emotions. Internal consistencies of each of the three emotion types calculated for each condition in each of the four cultural groups were reasonable (Cronbach's alphas for general positive emotions, disengaging positive emotions, and engaging positive emotions in the influence condition = 0.72, 0.80, and 0.80 in North Americans, 0.73, 0.68, and 0.87 in Hokkaido-born Japanese in Hokkaido, 0.60, 0.86, and 0.82 in mainland-born Japanese in Hokkaido, and 0.68, 0.84, and 0.91 in mainland Japanese; Cronbach's alphas for general positive emotions, disengaging positive emotions, and engaging positive emotions in the adjustment condition = 0.86, 0.85, and 0.76 in North Americans, 0.73, 0.68, and 0.87 in Hokkaido-born Japanese in Hokkaido, 0.72, 0.76, and 0.84 in mainland-born Japanese in Hokkaido, and 0.75, 0.71, and 0.90 in mainland Japanese). Although disengaging positive emotion and engaging positive emotion were highly correlated except for adjustment situations in Hokkaido-born Japanese in Hokkaido and mainland Japanese, they were still separate (0.01 < *r*s < 0.78). In each culture, the average intensity of the generally positive emotion over the five situations was regressed with the corresponding average intensity of the disengaging positive emotion and the corresponding average intensity of the engaging positive emotion in each condition. The standardized regression coefficients for disengagement and engagement in the influence and adjustment conditions are plotted in Figures 1, 2 respectively.

In the influence condition, for North Americans happiness was significantly predicted by disengaging positive emotion, β = 0.49, *t*_(21)_ = 2.50, *p* < 0.05, whereas the association between happiness and engaging positive emotion was relatively weak, β = 0.37, *t*_(21)_ = 1.89, *p* < 0.10. This same tendency was found in the two Hokkaido Japanese groups. For Hokkaido-born Japanese in Hokkaido, happiness was significantly predicted by disengaging positive emotion, β = 0.59, *t*_(16)_ = 2.50, *p* < 0.05, whereas it was not predicted by engaging positive emotion, β = 0.25, *t*_(16)_ = 1.08, *p* > 0.20. For mainland-born Japanese in Hokkaido, happiness was significantly predicted by disengaging positive emotion, β =0.48, *t*_(17)_ = 2.46, *p* < 0.05, whereas happiness was somewhat associated with engaging positive emotion, β =0.35, *t*_(17)_ = 1.80, *p* < 0.10. In contrast, for mainland Japanese happiness was significantly predicted by engaging positive emotion, β = 0.61, *t*_(24)_ = 3.77, *p* < 0.001, not by disengaging positive emotion, β =0.19, *t*_(24)_ = 1.14, *p* > 0.20.

In the adjustment condition, in the three Japanese groups happiness was significantly predicted by engaging positive emotion (Hokkaido-born Japanese in Hokkaido: β = 0.68, *t*_(15)_ = 3.64, *p* < 0.005; mainland-born Japanese in Hokkaido: β = 0.55, *t*_(15)_ = 2.31, *p* < 0.05, mainland Japanese: β = 0.70, *t*_(24)_ = 5.16, *p* < 0.0001), not by disengaging positive emotion (Hokkaido-born Japanese in Hokkaido: β = 0.12, *t*_(15)_ = 0.62, *p* > 0.50; mainland-born Japanese in Hokkaido: β = 0.25, *t*_(15)_ = 1.05, *p* > 0.30, mainland Japanese: β = 0.19, *t*_(24)_ = 1.37, *p* > 0.15). In contrast, for North Americans happiness was significantly predicted by disengaging positive emotion, β = 0.64, *t*_(20)_ = 3.41, *p* < 0.005, not by engaging positive emotion, β = 0.18, *t*_(20)_ = 0.94, *p* > 0.3^7^.

Finally, a multiple regression analysis including the culture and disengaging positive emotion and the culture and engaging positive emotion interactions was performed in each condition to see cultural differences in the associations of disengaging and engaging positive emotions with happiness. As shown in Table 4, neither the culture and disengaging positive emotion interaction nor the culture and engaging positive emotion interaction was significant due to relatively small sample size, except for a marginally significant interaction between culture and disengaging positive emotion when mainland Japanese and Hokkaido-born Hokkaido Japanese were compared in the influence condition. This effect indicates that the association between happiness and disengaging positive emotion for the Hokkaido-born Japanese in Hokkaido was somewhat greater than for the mainland Japanese.

**Table 4:** **Cultural differences in the associations between happiness and disengaging positive emotions and between happiness and engaging positive emotions for each condition (Study 2)**.

	**Culture × DEP**		**Culture × EP**	
	***t***	***p***	***t***	***p***
**CULTURE: NORTH AMERICANS = 1, MAINLAND JAPANESE = 0**				
Influence condition	1.33	0.19	−1.45	0.15
Adjustment condition	1.54	0.14	−1.48	0.15
**CULTURE: NORTH AMERICANS = 1, HOKKAIDO-BORN JAPANESE IN HOKKAIDO = 0**				
Influence condition	−0.66	0.51	0.33	0.74
Adjustment condition	1.58	0.12	−1.69	0.10
**CULTURE: NORTH AMERICANS = 1, MAINLAND-BORN JAPANESE IN HOKKAIDO = 0**				
Influence condition	−0.02	0.98	−0.29	0.77
Adjustment condition	1.52	0.14	−0.97	0.34
**CULTURE: HOKKAIDO-BORN JAPANESE IN HOKKAIDO = 1, MAINLAND JAPANESE = 0**				
Influence condition	1.87	0.07	−1.63	0.11
Adjustment condition	−0.31	0.76	0.66	0.51
**CULTURE: MAINLAND-BORN JAPANESE IN HOKKAIDO = 1, MAINLAND JAPANESE = 0**				
Influence condition	1.38	0.18	−1.00	0.32
Adjustment condition	−0.13	0.90	−0.18	0.86
**CULTURE: HOKKAIDO-BORN JAPANESE IN HOKKAIDO = 1, MAINLAND-BORN JAPANESE IN HOKKAIDO = 0**				
Influence condition	0.63	0.53	−0.54	0.59
Adjustment condition	−0.18	0.86	0.61	0.54

*DEP, disengaging positive emotion; EP, engaging positive emotion*.

Although cultural differences in the patterns on predictors of happiness were equivocal as in Study 1, Hokkaido-born Japanese in Hokkaido felt happier as they experienced more disengaging positive emotions when they influenced the surrounding people. The pattern was similar to that in North Americans. In contrast, they felt happier as they experienced more engaging positive emotions when they adjusted themselves to the surrounding people. The pattern was similar to that in mainland Japanese. In Hokkaido-born Japanese in Hokkaido, thus, the patterns of the predictors of happiness differed depending on whether they experienced either influence or adjustment situations.

As expected, for North Americans happiness was significantly predicted by disengaging positive emotions, whereas for mainland Japanese happiness was significantly predicted by engaging positive emotions. Moreover, as in Study 1, these tendencies emerged regardless of the types of situations. This may suggest that influence and efficacy are so powerful as cultural values in North America that they penetrate even adjustment situations, whereas adjustment and harmony as cultural values are so powerful in mainland Japanese culture that they penetrate even influence situations.

Furthermore, as in Study 1, the pattern of mainland-born Japanese in Hokkaido was similar to that of Hokkaido-born Japanese in Hokkaido. This is consistent with Kitayama et al. (2006a) showing that mainland-born Hokkaido Japanese were statistically no different from Hokkaido-born Hokkaido Japanese regarding the extent to which disengaging and engaging positive emotions predicted happiness.

## General discussion

The present research focused on Hokkaido, a northern island of Japan that underwent a history of voluntary settlement, and tested the hypothesis that interdependent culture of mainland Japan and independent culture associated with frontier coexist there. Examining the predictors of happiness in North Americans, mainland Japanese, and Hokkaido-born and mainland-born Japanese in Hokkaido, two studies demonstrated that in Hokkaido-born Japanese in Hokkaido, happiness was likely to be associated with disengaging positive emotions encouraged by achieving a personal goal through personal and influence behaviors. The pattern was more similar to that in North Americans than that in mainland Japanese. In contrast, in Hokkaido-born Japanese in Hokkaido, happiness was more predicted by engaging positive emotions encouraged by achieving an interpersonal goal through interpersonal and adjustment behaviors. The pattern was more similar to that in mainland Japanese than that in North Americans. The results are consistent with predictions that the ideas of independence and interdependence coexist in Hokkaido-born Japanese in Hokkaido, guided by the unique history of voluntary settlement which emerged in the mainland-Japanese predominant ethos of interdependence.

The present research suggests that the consequences of voluntary settlement are different, depending on the given cultural environment in which voluntary settlement occurred. The current findings would contribute to elaborating the voluntary settlement hypothesis (Kitayama et al., 2006a, 2009; Kitayama and Bowman, 2010). Previous findings showed that in spite of the shared Western cultural heritage, North Americans, who went through the history of voluntary settlement, are more independent than are Western Europeans (Kitayama et al., 2009). Thus, if voluntary settlement occurred in cultural context of independence, it would further enhance psychological tendencies motivated toward independence. On the other hand, the current findings suggest that if voluntary settlement occurred in the originally predominant ethos of interdependence, the independent and interdependent orientations would coexist and they would be internalized in people living therein.

Interestingly, the results of North Americans and mainland Japanese indicate that the patterns of the predictors of happiness tend to be stable regardless of the types of situations. Replicating the previous findings, happiness is likely to be associated with disengaging positive emotions in North Americans, whereas happiness is likely to be associated with engaging positive emotions in mainland Japanese. These results suggest that the effects of the types of situations (i.e., personal/influence vs. interpersonal/adjustment) are minimal, as long as these situations are produced in a single system of cultural practices, and consequently cultural values deeply penetrate in the situations. On the other hand, given the findings of situational priming (e.g., Kitayama et al., 1997; Morling et al., 2002; Tsai et al., 2007), it is no wonder that some situational effects in one's psychological tendencies could appear even in the two cultural groups. As with the previous studies of situational priming, if mainland Japanese are presented with typical personal/influence behaviors, for example ones described by North Americans, and imagine that they experienced the behaviors, or if North Americans are presented with typical interpersonal/adjustment behaviors, for example ones described by Japanese, and imagine that they experienced the behaviors, the patterns of the predictors of happiness might be influenced by the types of the behaviors.

Moreover, given a recent study by Miyamoto and Wilken (2010), which found that interpersonal influence augments analytic perceptual style in the US but not in Japan, if situational influences in the predictors of happiness appear, the influences may depend on the extent to which people are chronically oriented toward either independence or interdependence. Thus, an association between happiness and disengaging positive emotions in personal/influence behaviors may become prominent in Americans who are chronically oriented toward interpersonal influence, whereas an association between happiness and engaging positive emotions in interpersonal/adjustment behaviors may become prominent in mainland Japanese who are chronically oriented toward interpersonal adjustment. Exploring these speculations along a culture x person x situation approach (Cohen, 2007) may contribute to further understanding of the mutual relationship among culture, independent vs. interdependent orientations, and emotional experiences.

Consistent with findings by Kitayama et al. (2006a), the pattern on the predictors of happiness in mainland-born Hokkaido Japanese, who would be called “current settlers,” was similar to that in Hokkaido-born Hokkaido Japanese. The results support Kitayama et al. (2006a)'s suggestion that selection bias functions primarily on psychological propensities toward personal goal pursuit. On the other hand, the two Hokkaido groups are different in terms of their heritage culture whether they were born and brought up either in Hokkaido or in mainland Japan. Indeed, Kitayama et al. (2006a, Study 3) also found such an influence of initial enculturation regarding one's interpretation on others' behaviors. Hokkaido-born Hokkaido Japanese were likely to endorse a lay theory of dispositionism as did North Americans, whereas Mainland-born Hokkaido Japanese were likely to endorse a lay theory of situationalism as did mainland Japanese. Similarities and differences between these two Hokkaido groups should be examined further to understand a mechanism how the idea of independence fostered by voluntary settlement and the idea of interdependence penetrated in Japanese culture influence one's psychological tendencies.

The present research would provide an important insight into cultural change. The association between voluntary settlement and the ethos of independence would result from not only selection bias, but also an adaptation to harsh frontier ecological conditions and primitive social institutions. Thus, once cultural practices valuing independence like personal goal pursuits and personal choice were induced and established in new lands, they would be transmitted to children by child rearers providing feedback congruent with the cultural practices, and would be acquired by children through socialization. The process of transmission would contribute in sustaining the ethos of independence in Hokkaido. On the other hand, Hokkaido is completely integrated into mainland Japan. People living therein speak Japanese, watch Japanese national TV programs, and are familiar with the high-context communication practices in mainland Japan. Consequently, Hokkaido Japanese pay attention to paralinguistic cues like vocal tone as much as do mainland Japanese in comprehension of emotional utterances (Ishii, 2014). Thus, contemporary culture of Hokkaido is a hybrid of culture of mainland Japan and culture of frontier. Two issues might be considered regarding the process of cultural change. The first issue relates to the socialization process in Hokkaido and mainland Japan. If children's behaviors might be refashioned by child rearers' feedback facilitating ways of thinking and feeling that are preferable in a given culture, there might be some differences between Hokkaido and mainland Japan in what are praised or criticized in families and schools. In Hokkaido child rearers might be more likely to provide feedback that emphasizes personal goal pursuit and personal choice, compared to those in mainland Japan. The second issue relates to a possibility that the ethos of independence might fade and finally disappear in Hokkaido. Kitayama and Bowman (2010) proposed that voluntary settlement in Hokkaido highly fostered normative beliefs valuing independence (e.g., autonomy and self-reliance); nevertheless, it did not accompany an intrinsic motivation to achieve independence. Given this, behaviors of Hokkaido-born Japanese in Hokkaido might be regulated by one's belief that it may be socially beneficial to continue with the norm valuing independence. The ethos of independence might be sustained to some extent accordingly as long as such a belief exists.

Finally, there are some limitations to the current study, which should be taken into account. First, we conducted the current study for only undergraduates in Japanese and American universities. It would be necessary to test participants in a nationwide sample to confirm the validity of the current findings and generalize the effect of voluntary settlement fostering the ethos of independence. Second, although we prepared multiple situations in both studies, the number is relatively small. Although a preliminary analysis was attempted (see Footnote 4), this prevented us from examining potential differences between tendencies toward independence and interdependence and the moderation effect of types of situations within each participant. Thus, the present research could not address clearly either whether individuals in Hokkaido would be happier when they experienced disengaging positive emotions in personal behaviors and influence situations or whether they would be happier when they experienced engaging positive emotions in interpersonal behaviors and adjustment situations. This warrants further study including more situations. Third, past studies have proposed that the ethos of independence is associated with several socio-environmental factors including social class (e.g., Snibbe and Markus, 2005; Kraus et al., 2009), power (e.g., Miyamoto and Wilken, 2010), residential mobility (e.g., Oishi, 2010), daily economic activities (e.g., Uskul et al., 2008), and urbanism (e.g., Kashima et al., 2004; Yamagishi et al., 2012). Although we tried to make potential influences of these factors minimum by testing relatively homogeneous groups of undergraduates, it is still unclear whether the association between voluntary settlement and the ethos of independence is found independently of these alternative factors. Further examination is needed to determine the extent to which these socio-environmental factors including voluntary settlement foster the ethos of independence.

To conclude, the present research provided initial evidence that the consequences of voluntary settlement differ across cultures valuing independence vs. interdependence. Further investigation is needed to overcome the limitations of the current study and elaborate the voluntary settlement hypothesis, which would further our understanding of the mechanisms of cultural change and persistence. We believe, however, that the current evidence clearly highlights the psychological processes that are historically path dependent in lands of frontiers and, thus, deserves particular attention.

### Conflict of interest statement

The authors declare that the research was conducted in the absence of any commercial or financial relationships that could be construed as a potential conflict of interest.

## Footnotes

^1^The regions where mainland-born Japanese in Hokkaido were born and brought up were as follows: Tohoku (6), Kanto (22), Chubu (22), Kansai (8), Chugoku (4), Shikoku (2), and Kyushu (4). Yamagishi et al. (2012) classified Tokyo, Osaka, Kanagawa, Chiba, and Aichi prefectures as metropolitan areas. Based on it, 14 (Tokyo [4], Osaka [3], Kanagawa [1], Chiba [1], Aichi [5]) out of 68 mainland-born Japanese in Hokkaido were originally from metropolitan areas.

^2^While North Americans and the two groups of Hokkaido Japanese engaged in only the questionnaire used in Study 1, the data of mainland Japanese were collected through a part of a prescreening test to make a subject pool for other studies. Due to this different manner of data collection, data on participants' age and birth place in mainland Japanese were not accidentally collected.

^3^The average intensity for each participant was also computed for general negative emotions, disengaging negative emotions, and engaging negative emotions across situations. The average intensity for a generally negative emotion was regressed with the average intensity for disengaging negative emotion and that for engaging negative emotion for each situation in each culture. Overall, regardless of situations, the size of standardized regression was larger than 0 except for a few cases (for disengaging negative emotion, North Americans: 0.18 < βs < 0.76, Hokkaido-born Japanese in Hokkaido: −0.01 < βs < 0.60, mainland-born Japanese in Hokkaido: −0.04 < βs < 0.60, mainland Japanese: 0.36 < βs < 0.58; for engaging negative emotion, North Americans: −0.02 < βs < 0.64, Hokkaido-born Japanese in Hokkaido: −0.03 < βs < 0.86, mainland-born Japanese in Hokkaido: −0.06 < βs < 0.65, mainland Japanese: 0.12 < βs < 0.37). A 2 (situation) × 4 (culture) × 2 (emotion) ANOVA on the mean standardized regression coefficients did not show any significant effects, *p* > 0.10.

^4^Although associations between disengaging/engaging positive emotions and happiness across individuals were analyzed in the main text, it is unclear whether similar patterns of association can be observed within each individual. To determine it, following a procedure explained in Kitayama et al. (2006b) and Kitayama et al. (2009), for each individual, we initially computed standardized regression coefficients predicting happiness for disengaging and engaging positive emotions over all the six behaviors of personal situations and all the four behaviors of interpersonal situations, respectively. The standardized regression coefficients were then averaged over the participants in the two situations in the four cultural groups. Kitayama et al. (2006b) mentioned that the two steps “were simultaneously estimated with an algorithm provided by the hierarchical linear models (HLM) analysis” (p. 895). One hundred and eighty-six participants (7 North Americans, 42 Hokkaido-born Japanese in Hokkaido, 35 mainland-born Japanese in Hokkaido, and 102 mainland Japanese) were excluded in the following analysis because regression coefficients predicting happiness could not be computed due to missing values and a variable having no variance. A 2 (situation) × 4 (culture) × 2 (emotion) ANOVA on the mean standardized regression coefficients showed a main effect of emotion. Overall, one's happiness was associated more with engaging positive emotions (*M* = 0.67) than with disengaging positive emotions (*M* = 0.16), F_(1, 492)_ = 65.86, *p*< 0.0001. However, the effect was not qualified by either situation or culture, Fs < 1.5, ps > 0.20. The mean standardized regression coefficients in the two situations in the four cultural groups were as follows: For disengaging positive emotions in personal situations, disengaging positive emotions in interpersonal situations, engaging positive emotions in personal situations, and engaging positive emotions in interpersonal situations = 0.28, 0.27, 0.54, and 0.64 in North Americans, 0.09, 0.07, 0.74, and 0.66 in Hokkaido-born Japanese in Hokkaido, 0.20, 0.22, 0.59, and 0.67 in mainland-born Japanese in Hokkaido, and 0.14, 0.14, 0.56, and 0.81 in mainland Japanese.

^5^The regions where mainland-born Japanese in Hokkaido and mainland Japanese were born and brought up were as follows: Tohoku (6), Kanto (19), Chubu (6), Kansai (5), Chugoku (1), and Kyushu (1) for mainland-born Hokkaido Japanese, Tohoku (1), Kanto (3), Chubu (14), Kansai (32), Chugoku (1), Shikoku (1), and Kyushu (2) for mainland Japanese. Based on Yamagishi et al. (2012)'s criterion (see Footnote 1), 13 (Tokyo [5], Osaka [2], Kanagawa [3], Chiba [1], Aichi [2]) out of 38 mainland-born Japanese in Hokkaido and 17 (Tokyo [1], Osaka [11], Kanagawa [1], Aichi [4]) out of 54 mainland Japanese were originally from metropolitan areas. The proportion of participants who were originally from metropolitan areas was not significantly different between the two groups, chi-square (1) = 0.08, *p*> 0.70. Moreover, the proportion of mainland-born Hokkaido Japanese participants who were originally from metropolitan areas in Study 2 was not significantly different from that in Study 1 (14 out of 68), chi-square (1) = 2.38, *p* > 0.10.

^6^Tsai et al. (2007) suggests that influence goals are associated with high-arousal positive states (HAP), whereas adjustment goals are associated with low-arousal positive states (LAP). Among a series of emotions used in the current study, “elated” and “calm” are defined as HAP and LAP, respectively. Preliminary analysis showed a marginally significant interaction between type of situation (2: influence and adjustment) and type of positive state (elated and calm) on the average intensity reported by participants, *F*_(1, 168)_ = 3.25, *p* = 0.073. Participants in the influence situation reported elated (*M* = 3.01, *SD* = 1.08) more strongly than calm (*M* = 2.72, *SD* = 0.90), *t*_(168)_ = 2.71, *p* < 0.001, whereas the tendency disappeared in the adjustment situation (elated: *M* = 2.52, *SD* = 0.93, calm: *M* = 2.52, *SD* = 0.94). The affect-by-situation interaction was not qualified by a significant 3-way interaction with cultural group, *F* < 1. The situational differences on the intensity of experiencing the emotions suggest that the experimental manipulation was effective across cultures.

^7^For exploratory purposes, we also performed a similar regression analysis on general negative emotions. In the three Japanese groups, except for one case (mainland-born Japanese in the influence situation), unhappiness was significantly predicted by disengaging negative emotion, 0.48 < βs < 0.79, *p*s < 0.01, whereas it was not predicted by engaging negative emotion, −0.20 < βs < 0.28, *p* > 0.10, regardless of the types of situations. The tendency was also reported in Kitayama et al. (2006a). In two cases (mainland-born Japanese in the influence situation and North Americans in the adjustment situation), neither disengaging negative emotion (βs = 0.27 and 0.35, *p*s > 0.10) and nor engaging negative emotion (βs = 0.30 and 0.34, *p*s > 0.10) predicted unhappiness. In the remaining one (North Americans in the influence situation), unhappiness was significantly predicted by engaging negative emotion, β = 0.78, *t*_(21)_ = 5.34, *p* < 0.0001, whereas it was not predicted by disengaging negative emotion, β = 0.07, *t*_(21)_ = 0.49.
